# Sensory characteristics of high-amylose maize-resistant starch in three food products

**DOI:** 10.1002/fsn3.15

**Published:** 2012-12-20

**Authors:** Mindy Maziarz, Melanie Sherrard, Shanil Juma, Chandan Prasad, Victorine Imrhan, Parakat Vijayagopal

**Affiliations:** Department of Nutrition and Food Sciences, Texas Woman's UniversityPO Box 425888, Denton, Texas

**Keywords:** Dietary fiber, functional food, resistant starch, sensory evaluation

## Abstract

Type 2 resistant starch from high-amylose maize (HAM-RS2) is considered a functional ingredient due to its positive organoleptic and physiochemical modifications associated with food and physiological benefits related to human health. The sensory characteristics of three types of food products (muffins, focaccia bread, and chicken curry) with and without HAM-RS2 were evaluated using a 9-point hedonic scale. The HAM-RS2-enriched muffins, focaccia bread, and chicken curry contained 5.50 g/100 g, 13.10 g/100 g, and 8.94 g/100 g RS, respectively, based on lyophilized dry weight. The HAM-RS2-enriched muffin had higher moisture content and was perceived as being significantly moister than the control according to the sensory evaluation. The addition of HAM-RS2 to muffins significantly enhanced all sensory characteristics and resulted in a higher mean overall likeability score. The HAM-RS2-enriched focaccia bread appeared significantly darker in color, was more dense, and had the perception of a well-done crust versus the control. A grainer texture was observed with the chicken curry containing HAM-RS2 which did not significantly affect overall likeability. We concluded that the addition of HAM-RS2 may not significantly alter consumer's acceptability in most food products.

## Practical Application

This study shows that high-amylose maize (HAM-RS2) can completely or partially replace all-purpose (AP) flour in foods prepared commercially or in the home without significantly altering most sensory characteristics. The partial replacement of AP flour with HAM-RS2 in a medium-size muffin could provide 3.21 g of RS without impacting consumer acceptability. HAM-RS2 can completely replace AP flour in meat dishes with sauce or gravy to deliver 8.82 g RS per serving without influencing overall likeability. The functional ingredient HAM-RS2 can be used to increase dietary fiber content of certain foods with minimal impact on sensory characteristics.

## Introduction

Resistant starch (RS) has physical and biological properties that may benefit human health. RS is a functional fiber and comprises starch that is resistant to the digestive enzymes of the small intestine, therefore entering the large intestine intact (Englyst et al. 1992; Redgwell and Fischer 2005). Four types of RS have been classified: Type 1 is found in undamaged plant cell walls, such as seeds and legumes; Type 2 is a raw, native starch found in potatoes, green bananas, and high-amylose maize (HAM-RS2); Type 3 is retrograded starch formed during cooking mostly used as a food additive; Type 4 is chemically modified starch (Englyst et al. 1992; Sharma et al. 2008).

In the United States, adults consume an average of 4.9 g of RS per day, mostly from bread, cooked cereals and pastas, and vegetable sources (Murphy et al. 2008). A recommended daily intake of RS has not been established, but a minimum 14% of dietary starch as RS has been found to exert beneficial outcomes associated with gut health (Nugent 2005). Approximately 30–90% of RS entering the colon is fermented by gut microbiota to produce short-chain fatty acids, while the remainder is excreted (Sharma et al. 2008; Perera et al. 2010). RS is also classified as a prebiotic due to its ability to act as a substrate for beneficial gut microbiota, such as *Lactobacillaceae* sp., while inhibiting the colonization and growth of pathogenic species (Maathuis et al. 2009). In comparison with other dietary fibers, the bacterial fermentation of RS produces a higher percentage of butyrate and provides nutritional support to the epithelial cells of the colon (Kritchevsky 1995; Sharma et al. 2008). RS may also lower colon cancer risk. Bacterial fermentation of red meat protein in the colon can produce toxic, unfavorable by-products which may be involved in the development of cancer (Winter et al. 2011). Recently, Winter et al. (2011) found that adding 10% HAM-RS2 to a high-protein diet from red meat (40.9%) altered the bacterial fermentation patterns in the colon. This study found that the fermentation of HAM-RS2 was favored over protein, thus lowering cancer-causing by-products associated with red meat.

The physiological benefits of RS may have implications for the worldwide obesity epidemic. Partial replacement of flour with HAM-RS2 can lower the overall energy value of food products (Higgins 2004). Digestible starch provides approximately 4.2 kcal/g, while HAM-RS2 provides only 2.2–2.8 kcal/g (Behall and Howe 1996; Sharma et al. 2008). Ingesting HAM-RS2 has resulted in lower postprandial glucose concentrations and improvements in insulin sensitivity (Robertson et al. 2003, 2005; Higgins 2004; Yamada et al. 2005; Johnston et al. 2010; Ferrer-Mairal et al. 2011; Maki et al. 2012). Moreover, increased satiety was observed in young men 120 min after ingesting 50 g of HAM-RS2 (Anderson et al. 2010) and in men and women after consuming 48 g added to breakfast and lunch meals (Bodinham et al. 2010).

The US Dietary Reference Intakes suggest an adequate intake (25–38 g) of dietary fiber for disease prevention; however, only 15 g of fiber is consumed each day (Institute of Medicine, Food and Nutrition Board 2002; American Dietetic Association 2008). HAM-RS2, unlike other dietary fiber, can be added to foods as a means of increasing total dietary fiber intake without significantly altering the physiochemical properties of the fortified food (Korus et al. 2009). Certain types of fiber can produce a food product with undesirable characteristics and may appear dry, gritty, coarse, and negatively impact palatability (Fondroy et al. 1989; Fuentes-Zaragoza et al. 2010). However, the addition of HAM-RS2 is often desirable and may enhance the textural and sensory aspects of food products. HAM-RS2 is white and bland in flavor, consists of small-sized granules, and typically has a low water-holding but high water-binding capabilities (Sajilata et al. 2006). A study by Korus et al. (2009) found that replacing up to 20% of starch content with HAM-RS2 did not significantly change the organoleptic properties of gluten-free bread. Another study found no significant differences in the sensory characteristics “taste,” “overall acceptance,” and “consumption intention” in muffins when wheat starch was partially replaced with increasing percentages of HAM-RS2 (Baixauli et al. 2008a). The authors found that perceived sweetness and moisture were heightened with the addition of HAM-RS2 even though it has lower water-holding properties when compared with other dietary fibers. RS can also replace wheat flour (10% and 20%) in fried foods without compromising consumer acceptability (Sanz et al. 2008).

The purpose of this research was to examine the sensory characteristics of three food products after the partial or complete replacement of all-purpose (AP) and/or whole-wheat flour with HAM-RS2. The type of RS ingredient used in our products (Hi-maize® 260) is desirable because of its process stability in baking and storage (Nugent 2005).

## Materials and Methods

### Product and sample preparation

A type 2 RS product derived from high-amylose maize, containing approximately 60 percent RS and 40% amylopectin (Hi-Maize® 260), was provided by Ingredion International (Bridgewater, NJ).

Two formulations of blueberry muffins, herbed focaccia bread, and spicy chicken curry were created: RS and control. Each food formulation contained the same ingredients, as shown in Table 1, except Hi-maize fully or partially replaced the flour in the samples containing HAM-RS2. Sixty grams of Hi-maize replaced 98 g of AP and 30 g of whole-wheat flour in the muffin formulation to provide 4.73 g/100 g by total wet weight (tww), 36 g of Hi-maize replaced 36 g AP flour in the focaccia bread formulation to provide 10.17 g/100 g by tww, and 120 g of Hi-maize replaced 60 g of flour in the chicken curry formulation to provide 5.77 g/100 g by tww.

**Table 1:** Ingredient analysis of resistant starch (RS) and control formulations for muffins, focaccia bread, and chicken curry

	Ingredient (g/100 g)	
	
	RS formulation	Control
Muffins		
Hi-Maize®^1^	4.73	n/a
Banana, ripe	27.93	26.51
Flour, all-purpose	9.55	16.38
Blueberries, frozen	14.79	14.04
Sour cream, light	14.32	13.59
Brown sugar, light	12.84	12.19
Eggs, raw	7.89	7.49
Applesauce	4.38	4.16
Flour, whole wheat	2.37	4.49
Baking powder	0.59	0.56
Salt	0.24	0.22
Baking soda	0.18	0.17
Cinnamon, ground	0.15	0.15
Nutmeg, ground	0.04	0.04
Focaccia bread		
Hi-Maize®	10.17	n/a
Water	33.39	37.28
Mozzarella cheese	32.05	35.68
Flour, all-purpose	13.56	15.1
Olive oil	3.82	4.25
Vegetable oil	1.98	2.2
Active dry yeast	1.7	1.89
Salt	0.85	0.94
Parmesan cheese	0.71	0.79
Granulated sugar	0.59	0.66
Garlic powder	0.44	0.49
Oregano, dried	0.25	0.28
Thyme, dried	0.2	0.22
Basil, dried	0.1	0.11
Black pepper	0.1	0.11
Chicken curry		
Hi-Maize®	5.77	n/a
Yogurt, plain	35.34	43.46
Chicken breast, raw	32.71	30.04
Tomatoes, diced	16.36	15.02
Onion, chopped	7.93	7.28
Flour, all-purpose	n/a	2.65
Canola oil	1.01	0.93
Curry powder	0.45	0.42
Garlic, minced	0.43	0.4

1 Hi-Maize® 260 (a heat-moisture treated form of high-amylose maize supplied by Ingredion International, Bridgewater, NJ). Amount is based on total wet weight (tww) of each food product.

All recipes were prepared by culinary science students in the food preparation laboratory kitchen at Texas Woman's University. The muffin batter was prepared by combining light sour cream, light brown sugar, unsweetened applesauce and eggs into a bowl and mixed using a hand mixer at maximum speed for 1 min. The bananas were mashed and added. In a separate bowl, AP and whole-wheat flour, Hi-maize (RS muffins only), and other dry ingredients were combined and incrementally added to the wet mixture while mixing at medium speed for 2 min. Blueberries, thawed, were folded into the batter. Muffin pans were coated with nonstick spray and 60 g of batter were added to each well and baked in a conventional oven on the same rack for 23 min at 177°C. The muffins were cooled for 5 min, cut into quarters, and prepared for the sensory evaluation the same day. The focaccia bread was prepared by mixing the AP flour, Hi-maize (RS focaccia bread only), salt, sugar, yeast, and spices in a large bowl with a spatula. The canola oil and water were added and mixed with a spatula for 2 min. The dough was kneaded by hand on a floured surface for 3 min, covered with a damp cloth and allowed to rise for 20 min at 32°C. The dough was shaped into a ½-inch-thick rectangle, brushed with olive oil, parmesan and mozzarella cheeses, and baked on a baking sheet at 230°C for 15 min. Each loaf of focaccia bread was baked on the same oven rack and yielded eight slices. The RS and control chicken curry was prepared by sautéing minced garlic and canola oil in a sauté pan over medium heat for 1 min, and then diced tomatoes and curry powder were added and sautéed for 10 min. Boneless, skinless, raw chicken cut into ¼-inch cubes were added and cooked for 7 min. Onions, diced, were added and the mixture cooked for 10 min over medium heat. The mixture was poured into a bowl. AP flour or Hi-maize (RS chicken curry only) was added to the saucepan in which the chicken mixture was formulated, followed by low-fat, plain yogurt. The roux was whisked for 5 min over medium heat. The chicken mixture was added to the roux and cooked over low heat for 30 min. The chicken curry was transferred to a crock pot at low setting to maintain temperature during the sensory evaluation.

Five grams each of the muffin and focaccia bread batter and cooked chicken curry formulations were frozen at −4°C in polyethylene bags for analysis of nonresistant starch (NRS) and RS content. The samples were coded randomly, which were blinded by the research analyst. The sensory evaluations occurred the same day the food products were cooked.

### Sensory evaluation

Participants were recruited from the Texas Woman's University campus and were between 18 and 60 years of age. Approximately two-thirds of the participants were female. The study was conducted in a sensory laboratory with individual testing booths. The muffins (*n* = 37) and focaccia bread (*n* = 35) were evaluated on the same day and the chicken curry (*n* = 32) on a separate day. One batch of each RS and control food product was prepared and evaluated. Four-digit numbers were randomly assigned to each sample and the researchers conducting the sensory evaluation were blinded. The participants evaluated RS and control samples of each of the following: a one-quarter portion of a muffin; a one-third portion of a focaccia bread slice; a ¼ cup serving of chicken curry served with ⅛ cup steamed white rice. The muffins and focaccia bread were room temperature when evaluated; chicken curry was warm. The samples were placed randomly on a tray and the participants evaluated the samples in the order in which they chose. Water was also provided and the participants were asked to rinse their palate between tastings.

A 9-point hedonic scale was used to evaluate the sensory attributes of each sample as shown in Table 2. The attributes used to describe the muffins and focaccia bread was color, moisture/dryness, mouthfeel, density, crust (focaccia bread only), and overall likability. Appearance, aroma, mouthfeel, flavor, and overall likeability were the attributes used to describe the chicken curry.

**Table 2:** Description of sensory evaluation attributes based on a 9-point hedonic scale of three food products: muffins, focaccia bread, and chicken curry

Food product	Attribute	Description
Muffins	Color	1 = Dark brown; 5 = Golden brown; 9 = Light brown
	Moisture/dryness	1 = Dry; 5 = Moderately moist; 9 = Very moist
	Mouthfeel	1 = Chewy; 5 = Neutral; 9 = Brittle
	Density	1 = Dense; 5 = Neutral; 9 = Fluffy
	Overall likeability	1 = Dislike extremely; 5 = Like moderately; 9 = Like extremely
Focaccia bread	Color	1 = Dark brown; 5 = Just right; 9 = Too light
	Moisture/dryness	1 = Dry; 5 = Moderately moist; 9 = Very moist
	Mouthfeel	1 = Chewy; 5 = Neutral; 9 = Brittle
	Density	1 = Dense; 5 = Neutral; 9 = Fluffy
	Crust	1 = Under baked; 5 = Neutral; 9 = Well done
	Overall likeability	1 = Dislike extremely; 5 = Like moderately; 9 = Like extremely
Chicken curry	Appearance	1 = Dislike extremely; 5 = Like moderately; 9 = Like extremely
	Aroma	1 = Unpleasant; 5 = Neutral; 9 = Pleasant
	Mouthfeel	1 = Grainy; 5 = Neutral; 9 = Smooth
	Flavor	1 = Dislike extremely; 5 = Like moderately; 9 = Like extremely
	Overall likeability	1 = Dislike extremely; 5 = Like moderately; 9 = Like extremely

### Quantification of RS and NRS

The frozen food samples were thawed overnight at 4°C, halved, and transferred to a mixing bowl and blended for 5 min at maximum speed using a hand mixer, then poured into 125 mL flasks. The flasks were placed horizontally at −4°C and rotated 90° every 15 min to create a ⅛-inch shell along the sides. The samples were frozen (−80°C) for 2 h, then freeze-dried (LabCono Corporation, Kansas City, MO) for 23 h and ground to a fine powder consistency. Water content was determined indirectly (AACC International 2000).

The samples were analyzed in triplicate using a commercially available RS assay kit (Magazyme International Ireland Ltd, Wicklow, Ireland). NRS was determined by adding amyloglucosidase (3 U/mL) and pancreatic α-amylase (10 mg/mL) enzyme solutions to each sample (100 mg) in a glass tube. The samples were shaken horizontally (200 rpm) for 16 h in a 37°C water bath. The samples were removed and pellets of RS were formed by triplicate additions of 50% ethanol followed by vortexing for 30 sec and centrifugation (1500 *g*) for 10 min. The supernatants from each wash were collected and analyzed for soluble starch after adjusting the volume with 100 μmol/L sodium acetate buffer (pH 4.5). The RS pellet was resuspended in 2 mol/L KOH. d-Glucose content was determined by adding glucose oxidase/peroxidase reagent to the samples and absorbances were measured at 510 nm (Tecan Infinite® M200, Mannedorf, Switzerland) against the reagent blank. Total starch content is the sum of NRS and RS.

### Statistical analysis

Mean sensory attributes for each food sample were determined. Two-tailed *t*-tests compared the differences in mean sensory attribute scores between same food samples with and without HAM-RS2. Statistical significance was achieved at *P* ≤ 0.05. The analysis was performed using SPSS for Windows GradPack 18 (SPSS Inc, Chicago, IL).

## Results

### RS content

As shown in the recipe formulations (per 100 g of tww), 4.73 g of the HAM-RS2 ingredient was added to the RS muffins providing 2.84 g RS, 10.17 g HAM-RS2 was added to the RS focaccia bread providing 6.10 g RS, and 5.77 g HAM-RS2 was added to the RS chicken curry providing 3.46 g of RS (Table 1). Based on average serving sizes of 113 g for the muffin, 64 g for the focaccia bread, and 255 g for the chicken curry, these HAM-RS2-enriched foods would contain 3.21, 3.90, and 8.82 g of RS, respectively, per serving.

Based on lyophilized dry weight (ldw, Table 3), the RS muffins, focaccia bread, and chicken curry contained 5.50, 13.10, and 8.94 g RS per 100 g, respectively. Of the food items without HAM-RS2 (control), the focaccia bread had the highest amount of RS, 4.17 g/100 g ldw. The chicken curry contained 0.85 g RS/100 g ldw and the muffins had the lowest amount, 0.51 g RS/100 g ldw.

**Table 3:** Resistant, nonresistant, and total starch content in food products with and without resistant starch (RS) (control)^1^

Food product	RS (g/100 g)	Non-RS (g/100 g)	Total starch	Percent RS	Percent non-RS
Muffins					
Control	0.51	43.18	43.69	1.16	98.84
RS	5.50	32.09	37.59	14.63	85.37
Focaccia bread					
Control	4.17	48.08	52.24	8.67	92.02
RS	13.10	45.19	58.29	22.47	77.43
Chicken curry					
Control	0.85	19.42	20.28	4.39	95.79
RS	8.94	15.21	24.15	63.00	37.00

1 Data shown are based on lyophilized dry weight (ldw).

### Moisture content

The RS muffins and focaccia bread had 1.85% and 3.86% higher moisture content, respectively, than the samples without HAM-RS2. In contrast, the moisture content of the chicken curry without HAM-RS2 contained 8.62% more moisture than the RS chicken curry.

### Sensory evaluation

**Figure 1:**
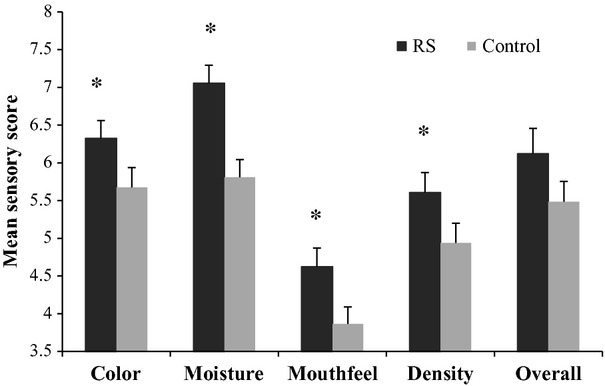
Mean sensory scores ± SE for resistant starch muffins and control. *Significant differences (*P* < 0.05) between the RS muffin and control.

**Figure 2:**
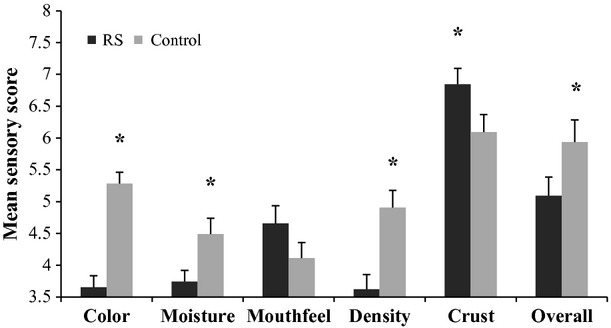
Mean sensory scores ± SE for resistant starch focaccia bread and control. *Significant differences (*P* < 0.05) between the RS focaccia bread and control.

**Figure 3:**
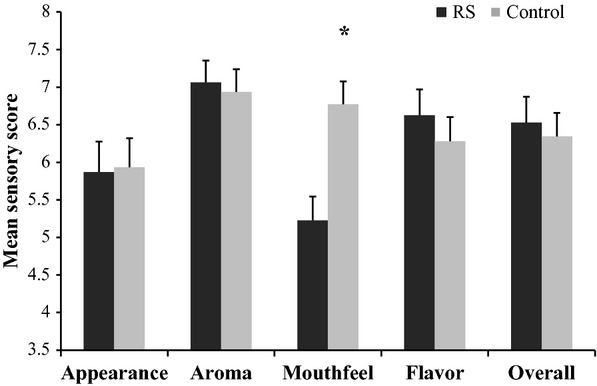
Mean sensory scores ± SE for resistant starch chicken curry and control. *Significant differences (*P* < 0.05) between the RS chicken curry and control.

A comparison of mean sensory attributes between food products with HAM-RS2 and the control is shown in Figures 1–3. The RS muffins were significantly lighter in color (*P* = 0.001), higher in moisture (*P* < 0.001), more brittle (*P* = 0.023), and less dense (*P* = 0.048) than the control, as shown in Figure 1. The RS focaccia bread was significantly darker (*P* < 0.001), drier (*P* = 0.025), denser (*P* < 0.001), and had a more well-done crust (*P* = 0.032) than the control, as shown in Figure 2. The RS focaccia bread had a significantly lower score for overall likeability (*P* = 0.030). Mouthfeel was the only significantly different sensory characteristic between the RS chicken curry and control, where the sample enriched with RS appeared grainier (*P* = 0.001, Fig. 3).

## Discussion

The results from our study indicate that the addition of HAM-RS2 can enhance the sensory properties of muffins. All sensory characteristics were significantly improved in the RS muffins when compared to the control, with the exception of overall likeability. Although overall likeability increased by 11.7%, it did not reach statistical significance. The RS muffins appeared significantly lighter in color than the control due to the white color of the HAM-RS2. Similar results were also observed in a study by Sanz et al. (2009) who compared the quality characteristics of muffins when wheat flour was replaced with different types of RS, including HAM-RS2. The HAM-RS2 muffin, which is the same type of RS used in this study, was significantly lighter in color than both RS Type 3 and control muffins. In addition, Baixauli et al. (2008b) reported that as the concentration of RS increased (0%, 5%, 10%, 15%, and 20%) in muffins, yellow and red hues were blunted. However, the human eye was not able to observe this effect until the concentration of RS in the muffins reached 15% or higher. The increased white appearance that can occur with the addition of HAM-RS2 has also been shown in other types of food products. Aigster et al. (2011) reported that granola bars and cereals containing 10% and 15%, respectively, of HAM-RS2 also had significantly higher L-value (whiteness) scores when compared with samples without RS.

The RS muffins in our study also scored significantly higher for density, appearing fluffier than the muffins without RS. The fluffiness of muffins correlates with the amount and size of air bubbles produced in the product during the baking process. Fluffier muffins can be perceived as a lighter, fresher product by consumers (Sanz et al. 2009). In addition, no significant differences in the “springiness” of HAM-RS2 muffins were found in the study by Sanz et al. Therefore, HAM-RS2 does not modify the overall fluffiness of baked product when compared with baked products without RS enrichment.

Many studies have shown the HAM-RS2 muffin did not result in significant differences in the sensory attributes “taste,” “overall acceptance,” or “consumption intention” when compared with a control (Baixauli et al. 2008a; Sanz et al. 2009), while our results implicate enhanced sensory characteristics of HAM-RS2-enriched muffins with no significant differences in overall likeability.

In our study, the RS focaccia bread was significantly darker in color, more dense, had a more well-done crust, and was less liked overall when compared with the control. Korus et al. (2009) also observed that increasing concentrations of HAM-RS2 in gluten-free bread produced a denser product, which could be explained by a reduction of yeast fermentation. The HAM-RS2 may act as “filler” and impact the production of CO_2_, resulting in a denser, less fluffy bread product. The authors concluded that HAM-RS2 in gluten-free bread did not significantly alter the organoleptic qualities, including “springiness,” “crumb hardness,” or “gumminess.” The significant difference in overall likeability of our focaccia bread is most likely due to the density and perception of a more well-done, or firmer, crust.

Our study found that the RS muffin had more moisture and scored significantly higher in the sensory attribute “moisture” than the control. This finding is in contrast to a study by Baixauli et al. (2008a) who compared the moisture content of muffins with increasing concentrations of HAM-RS2 (5%, 10%, 15%, and 20%). The authors found that high concentrations of HAM-RS2 resulted in significantly lower moisture content when compared with control muffins. Interestingly, the 10% HAM-RS2 muffins in their study had the lowest amount of moisture. The higher water content observed in our muffins could be due to the addition of whole-wheat fiber in the recipe. Other dietary fibers, especially wheat, have been found to have higher water-holding capacities (Rosell et al. 2009). Our study showed that the RS chicken curry had considerably less moisture than the control due to the complete substitution of flour with HAM-RS2. This indicates that the HAM-RS2 has lower water-holding capacity and higher evaporation capability than AP flour.

The sensory characteristics, including overall likeability of RS chicken curry, did not differ significantly from the control. The consumers rated the mouthfeel characteristic of the RS chicken curry as “grainier” with a mean sensory score of 5.23, lower than the control which had a mean score of 6.77. Similar results were found in a study examining the sensory attributes of HAM-RS2 in milk pudding. Our chicken curry recipe contained yogurt, a milk product with similar consistency to pudding. According to Ares et al. (2009), adding HAM-RS2 to milk pudding produced a significantly less acceptable product due to increased thickness and granular roughness. As the percentage of HAM-RS2 in the milk puddings increased above 1.4%, the acceptability of the product was hindered. Our RS chicken curry had a much higher concentration of HAM-RS2 (5.77% based on tww). The HAM-RS2 particle size averages 10–15 μm, and it has been reported that particles as small as 10 μm have been perceived in the mouth (Imai et al. 1995). Adding HAM-RS2 to roux or milk products may not be suitable for consumer acceptability, particularly when flour is completely replaced by HAM-RS2.

The amount of RS in the HAM-RS2-enriched food items (expressed as tww and ldw) varied due to differences in the moisture content for the muffins, focaccia bread, and chicken curry samples (56.33%, 45.79%, and 67.85%, respectively). According to the ldw analysis, the muffins and chicken curry had lower amounts of RS when compared with the tww calculation. In contrast, the focaccia bread had higher RS content in the ldw sample than tww calculation. The differences could be attributed to the lack of obtaining a homogenous product sample prior to RS determination.

Results from this research can be extrapolated to food items prepared commercially or in the home that may be enriched with HAM-RS2. A medium-size muffin (113 g) could provide 3.21 g of RS without significantly impacting the overall likeability. In addition, HAM-RS2 can also be incorporated into chicken dishes with sauce or gravy without influencing the overall likeability and can deliver up to 8.82 g of RS per serving (255 g).

Several limitations of this research should be reviewed. The RS content of the muffins and focaccia bread was analyzed using the raw, uncooked dough instead of the cooked product. The RS content of the chicken curry was analyzed using a cooked sample. However, this should not have altered the RS content in the products because the type of RS (a heat-moisture-treated form of HAM-RS2) used in our study is not changed during baking or storage (Nugent 2005). The participants recruited for the sensory analysis were not trained in sensory evaluation techniques; however, the data presented are likely representative of everyday consumer's acceptability of HAM-RS2-enriched products.

## Conclusions

The partial replacement of flour with HAM-RS2 in muffins enhanced the sensory characteristics without affecting overall likeability. HAM-RS2 in muffins created a product that appeared more moist than the control. The RS focaccia bread was perceived as having a more well-done crust and was denser than the control, which may have contributed to the lower overall likeability score. The addition of HAM-RS2 in roux or milk products could be desirable if other ingredients were able to mask the mouthfeel that can be attributed to RS. Overall, the addition of HAM-RS2 in foods may not significantly alter consumer's likeability and can be added as a functional fiber to promote physiological health-related benefits.
